# Development of Pineapple Microsatellite Markers and Germplasm Genetic Diversity Analysis

**DOI:** 10.1155/2013/317912

**Published:** 2013-08-19

**Authors:** Suping Feng, Helin Tong, You Chen, Jingyi Wang, Yeyuan Chen, Guangming Sun, Junhu He, Yaoting Wu

**Affiliations:** ^1^Bioscience and Biotechnology College, Qiongzhou University, Sanya 572200, China; ^2^Institute of Tropical Bioscience and Biotechnology, Chinese Academy of Tropical Agricultural Science, Haikou 571101, China; ^3^Institute of Tropical Crop Variety Resources, Chinese Academy of Tropical Agricultural Sciences, Danzhou 571737, China; ^4^South Subtropical Crops Research Institute, Chinese Academy of Tropical Agricultural Sciences, Zhanjiang 524091, China

## Abstract

Two methods were used to develop pineapple microsatellite markers. Genomic library-based SSR development: using selectively amplified microsatellite assay, 86 sequences were generated from pineapple genomic library. 91 (96.8%) of the 94 Simple Sequence Repeat (SSR) loci were dinucleotide repeats (39 AC/GT repeats and 52 GA/TC repeats, accounting for 42.9% and 57.1%, resp.), and the other three were mononucleotide repeats. Thirty-six pairs of SSR primers were designed; 24 of them generated clear bands of expected sizes, and 13 of them showed polymorphism. EST-based SSR development: 5659 pineapple EST sequences obtained from NCBI were analyzed; among 1397 nonredundant EST sequences, 843 were found containing 1110 SSR loci (217 of them contained more than one SSR locus). Frequency of SSRs in pineapple EST sequences is 1SSR/3.73 kb, and 44 types were found. Mononucleotide, dinucleotide, and trinucleotide repeats dominate, accounting for 95.6% in total. AG/CT and AGC/GCT were the dominant type of dinucleotide and trinucleotide repeats, accounting for 83.5% and 24.1%, respectively. Thirty pairs of primers were designed for each of randomly selected 30 sequences; 26 of them generated clear and reproducible bands, and 22 of them showed polymorphism. Eighteen pairs of primers obtained by the one or the other of the two methods above that showed polymorphism were selected to carry out germplasm genetic diversity analysis for 48 breeds of pineapple; similarity coefficients of these breeds were between 0.59 and 1.00, and they can be divided into four groups accordingly. Amplification products of five SSR markers were extracted and sequenced, corresponding repeat loci were found and locus mutations are mainly in copy number of repeats and base mutations in the flanking region.

## 1. Introduction

Pineapple (*Ananas comosus* (L.) Merr.), belonging to Bromeliaceae, ananas, is a perennial evergreen herbaceous fruit tree that produces one of the most famous four tropical fruits beside banana, coconut, and mango. During cultivation and propagation, due to the different naming habits of the propagators and local cultivators, homonym and synonym are very common, nomenclature of pineapple was in chaos, and breeds vary greatly within major groups, which not only hinders rational use of pineapple germplasm resources, but also impedes breeding of better pineapple strains.

Molecular marker technology, such as RFLP, RAPD, and AFLP, has been reported to be used in pineapple germplasm analysis; for example, Duval et al. [1] used RFLP marker in research on germplasm diversity of pineapple. De Fátima Ruas et al. [2] analyzed 18 germplasms of pineapple using RAPD marker and concluded that the cultivated germplasms in this study had a similarity coefficient lower than 0.85. Duval et al. [3] determined pineapple chloroplast DNA polymorphism using RFLP analysis. Kato et al. [4] analyzed intraspecific DNA polymorphism of pineapple using AFLP assay. Popluechai et al. [5] assessed genetic diversity of nine germplasms of pineapple and divided them into three groups based on a 0.77 similarity coefficient. Wöhrmann and Weising [6] developed EST-SSR markers to carry out cross-amplification study within the pineapple bromeliad species, genus, and subfamily. Their results have shown that most genetic markers had low polymorphism, especially when the subjects are closely related. The recently developed microsatellite marker attracts a lot of interests and is being widely used due to its comparatively high polymorphism and genome specificity [7].

SSR markers can be detected by PCR amplification using specific primers which can be developed mainly by classical library screening [8], microsatellite enriching [9, 10], 5′-anchoring PCR technology [11], sequence tagged microsatellite profiling (STMP) [12], selectively amplified microsatellite (SAM) [13], and bioinformatics methods [6, 14, 15]. Among these methods, SAM can generate SSR markers generating multilocus SSR fingerprints, which requires only one pair of primers and has high efficiency in developing informative SSRs. In this study, we designed SSR primers using SAM or bioinformatics method. Those highly informative and reproducible SSR primers were used to carry out germplasm diversity analysis for 48 breeds of pineapples, so as to reveal the genetic relationship among them, provide reference for improvement of the current chaotic situation of pineapple nomenclature, and reveal the regularity of mutation of pineapple SSR loci through amplification, extraction, and sequencing of SSR loci.

## 2. Materials and Methods

### 2.1. Materials

The Tainong 17 pineapple was used to develop SSR markers; materials for genetic diversity analysis were obtained from Institute of Tropical Crop Variety Resources and South Subtropical Crops Research Institute, Chinese Academy of Tropical Agricultural Sciences (Table 1). DNA was extracted using a modified CTAB method [16]. *E. coli* strain DH5*α* for transformation was kept by our laboratory.

### 2.2. Development of Genomic SSR Markers

Genomic library was constructed in reference to the SAM method [13]. *Pst*I (15 U/*μ*L, 0.3 *μ*L), *Mse*I (10 U/*μ*L, 0.5 *μ*L), 10x NEB buffer II (5 *μ*L), and BSA (10 *μ*g/*μ*L, 0.5 *μ*L) were added to 1 *μ*g of genomic DNA. Reaction was allowed at 37°C for 1 h and terminated by incubation at 65°C for 10 min. 5 pmol *Pst*I adaptor and 50 pmol *Mse*I adaptor were then added and incubated at 45°C for 5 min; then T4 DNA ligase (0.5 U), dATP (100 mM, 1.8 *μ*L), and sufficient reaction buffer were added to reach a total volume of 30 *μ*L, and the system was incubated at 16°C for 12 h for ligation. Product of SAM-PCR was separated using denaturing polyacrylamide gel electrophoresis. Based on Hayden and Sharp's [13] work, we increased the number of adaptors, sequences of adaptors, and primers used in this study are shown in Table 2. Target sequences were extracted, cloned, and sequenced, then screened for SSR sequences using the Microsatellite software (MISA) (http://pgrc.ipk-gatersleben.de/misa/), the criteria for SSR screening were as follows: mononucleotide must be repeated for 10 or more times, dinucleotide and trinucleotide be repeated for six or more times, and ≥4 nucleotide units be repeated for five or more times. Complicated SSRs that are interrupted by no more than 100 bases were also included. Dinucleotide repeats such as AT/TA, CT/AG were regarded as the same type.

Cluster analysis was carried out using stackPACK v 2.2 program [17]. Primers were designed using RIMER5.0 [18] and the main parameters were GC content, 40%–60%, annealing temperature, 48–60°C; anticipated product length, 100–300 bp. The primers were synthesized by Invitrogen.

20 *μ*L PCR reaction system consists of 10x PCR buffer, MgCl_2_ (1.5 mM), dNTPs (0.25 mM), forward/backward primers (5 pmol each), DNA template (20 ng), and Taq DNA polymerase (0.15 U); reaction program was predenaturation at 94°C for 3 min, 30 cycles of denaturation-annealing-elongation (94°C for 30 sec, 55°C for 45 sec, and 72°C for 1 min), and a final elongation at 72°C for 7 min. PCR product was separated using 8% nondenaturing polyacrylamide gel electrophoresis and visualized by silver staining.

### 2.3. Development of EST-SSR Markers

ESTs were obtained from the dbEST database of NCBI (http://www.ncbi.nlm.nih.gov/projects/dbEST) registered before February 2012.

ESTs that had PolyT or polyA (≥5 repeats) within 50 bp downstream of 5′-end or upstream of 3′-end or shorter than 100 bp were excluded using the EST-trimmer software (http://pgrc.ipk-gatersleben.de/misa/download/est_trimmer.pl); for ESTs that were longer than 700 bp, only the first 700 bp at the 5′-end were kept. Then, SSRs were screened using the MISA software. Screening criteria were the same as genome-based development.

Cluster analysis was carried out using stackPACK. Design and synthesis of primers were the same as genome-based development.

### 2.4. Genetic Diversity Analysis and of Locus Mutation Detection

Eighteen pairs of these newly developed primers that were highly informative and reproducible were selected to carry out genetic diversity analysis for 48 breeds of pineapple. After silver staining, electrophoresis bands were recorded using the Banscan software, for the same migration distance, positive band was recorded as “1,” negative band as “0,” and failure of amplification as “9.” Genetic distance matrix was calculated using NTSYSpc ver 2.1 software (http://www.exetersoftware.com/), evolutionary tree was constructed using the Unweighted Pair Group Method with Arithmetic Mean (UPGMAM) method, primer polymorphism informativeness was calculated using the formula PIC = 1 − ∑(*P*_*i*_)^2^, wherein *P*_*i*_ stands for the frequency of *i*th locus in all alleles [21].

Repeat types, (CA) *n*, (GCAGGA) *n*, (AG) *n*, (TCGCAG) *n*, and (TCT) *n* primers were used to amplify 10 samples that included bands corresponding to all the previous five repeat types; the bands were then recovered, sequenced, and subjected to SSR locus mutation analysis. The ClustalX software was used to compare original sequence and sequencing results.

## 3. Results

### 3.1. Development of Genomic SSR Markers

Products of SAM PCR were separated using denaturing polyacrylamide gel, and 200–750 bp bands were recovered after silver staining. A total of 99 bands were cloned and sequenced, 86 of them contained SSR loci. Numbers of bands obtained by combination of different anchoring primers and adaptor primers were shown in Table 3. Clustering analysis revealed 68 single sequences, and eight groups of repeated sequences; that is, a total of 76 sequences can be used for primer designing. *Pst*I SAM primer in combination with 5′ anchoring primer PAC/PGT developed 44 sequences and *Pst*I SAM primer in combination with 5′ anchoring primer PCT/PGA developed 55 sequences, indicating that CT/AG is more abundant than AC/GT in pineapple genome (Table 3). All sequences were screened for SSR loci using MISA; 52 GA/CT repeat loci were found and 39 AC/GT repeat loci were found, which is in accordance with the result developed by different anchoring primers. Three mononucleotide repeats were found and no tri- or more nucleotide repeat locus was found.

Thirty pairs of primers flanking the SSR locus were designed for each of the 36 SSR-containing DNA sequences; 24 of them generated clear, reproducible bands of expected size, and 13 of them showed polymorphism when amplifying the selected samples.

### 3.2. Development of EST-SSR Markers

**Figure 1:**
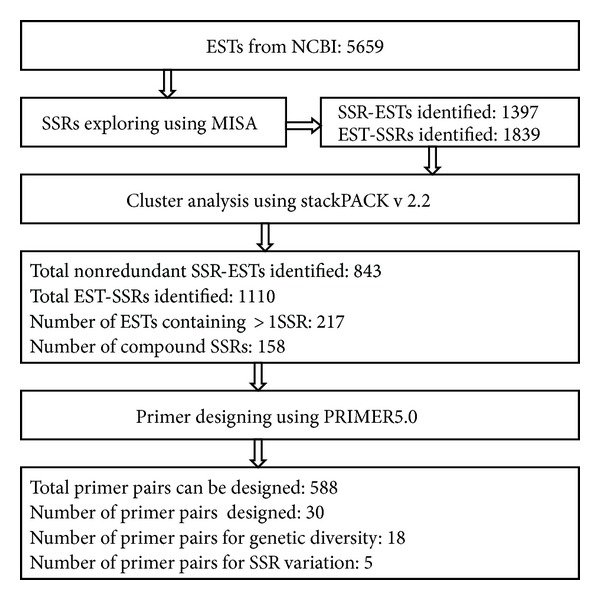
Scheme used for data exploring and development of EST-SSRs markers.

Fifty-six hundred and fifty-nine EST sequences with a total length of 4,141.084 kb were downloaded from NCBI database. MISA was used to analyze these sequences and 1397 EST sequences containing 1839 microsatellite loci were developed (Figure 1). Frequency of SSR-containing sequences among all sequences was 24.68% (one SSR locus every 4.05 ESTs) or one microsatellite locus every 2.25 kb.

Eight hundred and forty-three nonredundant SSR-containing EST sequences were obtained after cluster analysis on the 1397 EST sequences using stackPACK v 2.2. 620 of them were single sequence and 223 were redundant groups. 1110 SSR loci were identified with MISA, and 217 of these sequences contained more than one SSR locus. Of the 1110 SSR loci, 952 were simple SSRs, and 158 were complicated.

Frequencies of nonredundant EST-SSRs in pineapple ESTs were 1SSR/3.73 kb; most of them were small repeating units; taken away mononucleotide repeats, there were 381 (34.3%) dinucleotide repeats, mostly AG/CT accounting for 83.5%, followed by AT/AT accounting for 10.2%, AC/GT accounted for 6.0%, and CG/CG appeared only once; 158 (14.2%) trinucleotide repeats were found, mostly AGC/GCT (24.1%) and AAG/CTT (20.9%); 23 (2.1%) 4-nucleotide repeats were found, mostly AAAG/CTTT, 12 (1.1%) 5-nucleotide repeats were found, 33.3% of them were AAAAG/CTTTT; and 14 (1.3%) AAAAAG/CTTTTT 6-nucleotide repeats were found. In total, 44 types of SSRs were found (Table 4).

Of the 1110 SSR loci identified, taken away the 522 mononucleotide repeats, the other 588 EST-SSRs can be used for primer designing. Thirty pairs of primers were designed for each of 30 randomly selected EST-SSRs; 26 of them generated clear, reproducible bands, and 22 of them showed polymorphism.

### 3.3. Genetic Diversity Analysis and Detection of Mutation Locus

**Figure 2:**
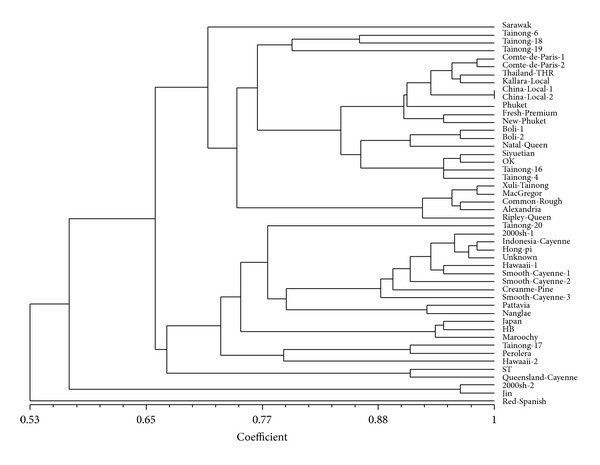
Dendrogram of pineapple varieties based on 18 SSRs primer pairs.

Eighteen pairs of highly informative primers developed by EST-SSR or from genomic library were selected to carry out PCR amplification and genetic diversity analysis for 48 breeds of pineapples (Table 5). The results showed that these 48 germplasms of pineapples had similarity coefficients between 0.59 and 1.0. Based on a similarity coefficient of 0.66, they were divided into four groups: Group 1 containing Sarawak, Tainong-6, Tainong-18, Tainong-19, Comte de Paris 1, Comte de Paris 2, Thailand THR, Kallara local, China Local 1, China Local 2, Phuket, Fresh Premium, New Phuket, Boli 1, Boli 2, Natal Queen, Seiyuetian, OK, Tainong-16, Tainong-4, Xuli Tainong, MacGregor, Common Rough, Alexandria and Ripley Queen; Group 2 containing Tainong-20, 2000sh 1, Indonesia cayenne, Hongpi, Unknown, Hawaii 1, Smooth cayenne 1, Smooth cayenne 2, Creanme pine, Smooth cayenne 3, Pattavia, Nanglae, Japan, HB, Maroochy, Tainong-17, Perolera, Hawaii 2, ST, and Queensland Cayenne; Group 3 containing 2000sh 2 and Jin; and Group 4 containing only Red Spanish (Figure 2). 

**Figure 3:**
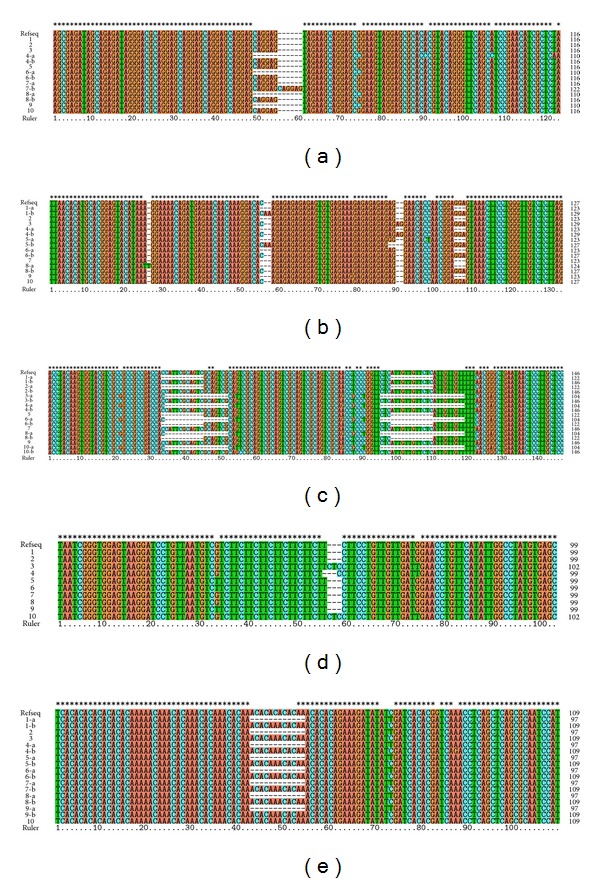
Sequences obtained using five SSRs markers amplifying across *Ananas comosus*. (a), (b), (c), (d), and (e) represent the marker EP-11, EP-12, EP-15, EP-20, and Bp-01, respectively. RefESTs in (a), (b), (c), and (d) represent the accession numbers: DT338752.1, DT338506.1, DT338171.1, and DT337383.1 in NCBI database, respectively. (e) represents AC1.3. The suffixes “a” and “b” represent the allele numbers. 1–10 represent: 1—Tainong-17, 2—Japan, 3—Comte de Paris, 4—Tainong-18, 5—Red Spanish, 6—MacGregor, 7—Jin, 8—Alexandria, 9—Pattavia; and 10—Phuket.

Five pairs of primers were used for recovery and sequencing, of which four were developed from EST-SSR, and the other one was developed from genomic library. After PCR amplification and sequencing, these five pairs of primers generated 73 sequences, different SSR markers generated corresponding sequences after amplification and sequencing. Through comparison by ClustalX software, insertions, deletions, transversions, and conversions of these SSR loci and flanking sequences were revealed (Figure 3).

## 4. Discussion

### 4.1. Efficiency of SAM Method to Develop SSR Markers

This study used the SAM method invented by Hayden and Sharp [13] to develop positive clones from pineapple genome for sequencing. Ninety nine clones were sequenced and 86 of them contained 94 SSR loci. Thirty-six of these sequences were selected, and 36 pairs of primers flanking the SSR loci were designed, one for each, and 24 of them generated clear and reproducible bands of expected size, and 13 of them showed polymorphism when amplifying selected samples. 86.9% of all sequenced clones were positive, and frequency of SSR marker showing polymorphism was 13.1%, which is lower than [22] results for rubber trees (24.6%) and Wang et al. [23] for banana (19.5%). This may be due to variations between different materials; although SSRs are widely distributed in eukaryotic genomes, their content, type, and copy number vary between different materials. Even within the same species, there would also be variances. Another modification was that primers were designed on repeating sequences of microsatellite, and only a portion of flanking sequence was used instead of the whole initial 5′ anchoring primer. This may have elevated reproducibility of the primers but may lower their polymorphism. Comparatively, the SAM method is much more efficient in developing SSRs than conventional constructing and screening from genomic library of small inserts or STMS method. For example, Ujino et al. [8] acquired only three positive SSR-containing sequences out of 6000 clones (0.05%) using conventional method, and Rajora et al. [24] developed 71 positive clones out of 4028 (1.8%) using STMS method.

### 4.2. SSR Sequence Analysis

All sequences were screened for repeat loci using MISA, AC/GT repeats accounted for 41.5%, GA/TC repeats accounted for 55.3%, which was in consistence with results acquired by different anchoring primers. Mononucleotide A/T repeats occurred three times (3.2%) no ≥3 nucleotide repeats were found. Relatively fewer repeat types were obtained in comparison with Rivera et al. [25], Viruel and Hormaza [26]. Such phenomenon can be explained by the following facts: first, the choice of length and type of the additional 3′ bases of preamplification primers reduced SSR productivity at the same time of reducing complexity of the template [13]; second, the parameters set for repeat screening also have certain effects; for example, we have set that 6-nucleotide must repeat five or more times and there was no such loci, but if the parameter was changed to four or more times, there would be one CAAACA/TGTTTG repeat; third, choice of probes may influence frequency of corresponding repeats; for example, Rajora et al. [24] used oligonucleotide sequences corresponding to different SSRs and the resulted SSRs had similar repeat units to the probes.

### 4.3. Comparison of the Development of Genomic SSR and EST-SSR

EST-SSR marker has unique advantages [27], including being able to detect polymorphism of expressing regions of the genome, high versatility, and relatively low development cost. Thus, it is of great value in genetic mapping, diversity of genetic resources, discovery, and positioning of functional genes, researches on origin of species, evolution, and genomic comparison [28].

Wöhrmann and Weising [6] screened NCBI database for SSRs, setting the criteria as no less than 15 times for mononucleotide repeats; no less than seven times for dinucleotide repeats, and no less than five times for 3–6 nucleotide repeats. Forty-two types SSRs were revealed from 5659 ESTs; one SSR occurred every 4.1 kb on average. Trinucleotide repeats was the most common, followed by dinucleotide repeats. Ong et al. [19] also developed SSR markers from 5931 ESTs using SynaRex tool. To ensure comparability between EST-based and genome-based SSR development, different analytic software and the same criteria for SSR screening were used in this study. For EST-based SSR marker development, due to differences in size of database, criteria for SSR screening, and tools for SSR development, the distribution, frequency, and abundance of SSRs also vary (Table 6), as concluded by Varshney et al. [28].

In this study, the rate of polymorphic SSR marker was 13.1% for SAM-developed SSRs, and 2.1% for EST-developed SSRs, showing a higher efficiency of SSR development by SAM method than genomic library-based method which was in consistence with results reported for soybean [29, 30] and rubber trees [22, 31].

### 4.4. Genetic Diversity Analysis for Pineapple Germplasms Using SSR Markers

In the present study, 48 germplasms of pineapple were divided into four subgroups, instead of three (Caine, Queen, and Spain) by conventional morphological classification. It can be observed from the cluster analysis, of the 25 germplasms in the first group, Kallara local, Phuket, New Phuket, Natal Queen, MacGregor, Common Rough, Alexandria and Riply Queen, and so forth, belong to the Queen group, and the others such as Tainong-6, Tainong-18, Tainong-19, Boli 1, Boli 2, Tainong-16 and Tainong-4 were the hybridizations of Caine and Queen, and so forth. The germplasms in the second group are morphologically divided into the Caine group, while Indonesia cayenne, Hawaii 1, Smooth cayenne 1, Smooth cayenne 2, Smooth cayenne 3, Pattavia, Nanglae, Hawaii 2, and Queensland Cayenne, and so forth, are hybridizations of Caine and Queen; the fourth group only contains the Red Spanish germplasm of the Spain group.

Some taxonomists regarded Perolera as a new breed, and in this study, it was clustered into the second group, which is closely related to the Caine group. The Sarawak germplasm was thought to belong to the Caine group, but it was actually clustered into the Queen group. This phenomenon may be due to non-unified classification standards for pineapple that leads to different classification; the name for the germplasm's confusion, for exemple, one germplasm has multiple names or a single name used by multiple germplasms because of frequent regional and international exchange of pineapple germplasms; the internal limitation of morphological classification that characteristics of a germplasm is easily affected by environmental conditions. During cultivation and propagation, due to different naming habits of the propagators and local cultivators, homonym and synonym are very common, nomenclature of pineapple was in chaos, and germplasms vary greatly within major groups. In addition, SSR reveals not only genetic variations at the DNA level, but also differences in genotype between germplasms. Genome DNA contains not only structural genes, but also some silence genes which had yet no clear function, and the perceptible phenotype is the results of functional gene expression under influence of both internal and external environment. So, difference in DNA structure may not necessarily lead to differences in morphology.

### 4.5. Analysis of SSR Mutation

Mutation of SSR mainly came from base changes in the flanking sequence and repeat region. In this study, we found no insertion or deletion mutations at the EP-11 or EP-20 loci, at the EP-12 locus, Alexandria-a, Tainong-17-b, and Red Spanish-b had “T” and “AA” insertion, respectively; flanking sequence of Bp and EP had deletions (Figure 3).

Flanking sequence of corn had insertion mutations [32]. Gutierrez et al. [33] found in their research on *M. truncatula* that sequence variation was mainly due to variation in copy number of repeats of the SSR region, as well as insertion, deletion, and nucleotide substitution mutations. Symonds and Lloyd [34] pointed out that interruptions in the SSR region shortened the SSR sequence; in this study, it was observed that nucleotide substitution resulted in decrease in copy number of repeats; a single long repeat sequence was divided into several smaller repeat sequences or became shorter. For example, the Golden pineapple had a CAGGAG insertion at the EP-11 “b” locus, increasing repeat number; the “T” of EP-20, Tainong-18 sequence was replaced with “C,” leading to decrease in TCT repeats; Red Spanish-b had its “A” replaced with “G” at the EP-12 locus and was thus divided into smaller repeating units.

## Figures and Tables

**Table 1:** Pineapple materials included in the study.

Name	Remarks	Seientifiename	Remarks
Sarawak		OK	
Tainong-6	Yellow Mauritius × Cayenne	Unknown	
Tainong-20		Creanme Pine	
Tainong-17	Cayenne♀ × Rough♂	China Local 2	
Xuli-Tainong		Common Rough	
Japan		Natal Queen	
2000sh 1		Queensland Cayenne	
2000sh 2		Riply Queen	
Tainong-19	Cayenne♀ × Rough♂	MacGregor	
Indonesia Cayenne		Jin	
HB		Maroochy	
Comte de Paris 1		Fresh Premium	
Comte de Paris 2		Perolera	
Boli 1	Cayenne♀ × Queen♂	Alexandria	
Thailand THR		Kallara Local	
ST		Smooth Cayenne 1	
Tainong-18		Pattavia	
China Local 1		Nanglae	
Siyuetian		Smooth cayenne 2	
Hawaii 1		New Puket	
Red Spanish		Phuket	
Hongpi		Smooth Cayenne 3	
Tainong-16	Cayenne♀ × Rough♂	Hwaaii 2	
Boli 2	Cayenne♀ × Queen♂	Tainong-4	Cayenne♀ × Queen♂

Chinese Academy of Tropic Agricultural Science, Danzhou.

**Table 2:** Sequences of the adapters and primers.

Name of primers	Sequences of primers (5′-3′)
*Pst*I adapter	Sense strand: CTC GGA AGC CTC AGT CCC AGA CTG CGT ACA TGC A-OH
	Antisense strand: phos-TGT ACG CAG TCT GGG ACT GAG GCT TCC GAG A-OH

*Mse*I adapter	Sense strand: GAG CAA GGC TCT CAC AAG GAC GAC CGA CGA G-OH
	Antisense strand: phos-TAC TCG TCG GTC GTC CTT GTG AGA GCC TTG CT-OH.

*Mse*I suppressed amplification primer	GAG CAA GGC TCT CAC A

*Pst*I suppression amplification primer	CTC GGA AGC CTC AGT C

*M*s*e*I preamplification primer	GAC GAC CGA CGA GTA AC

*Pst*I preamplification primer	AGA CTG CGT ACA TGC AGG A

*Pst*I SAM primers	Adapter 1: AGA CTG CGT ACA TGC AGG ACC
	Adapter 2: AGA CTG CGT ACA TGC AGG ACG
	Adapter 3: AGA CTG CGT ACA TGC AGG AGC
	Adapter 4: AGA CTG CGT ACA TGC AGG A CT
	Adapter 5: AGA CTG CGT ACA TGC AGG A TC
	Adapter 6: AGA CTG CGT ACA TGC AGG A CA
	Adapter 7: AGA CTG CGT ACA TGC AGG A AC
	Adapter 8: AGA CTG CGT ACA TGC AGG A GT
	Adapter 9: AGA CTG CGT ACA TGC AGG A TG
	Adapter 10: AGA CTG CGT ACA TGC AGG A GA
	Adapter 11: AGA CTG CGT ACA TGC AGG A AG
	Adapter 12: AGA CTG CGT ACA TGC AGG A AT

5′-Anchored SSR primers	PAC: a: KKR YRY YAC ACA CAC ACA C
	b: KKY RYR YCA CAC ACA CAC A
	PCT: a: KKV RVR VCT CTC TCT CTC T
	b: KKR VRV RTC TCT CTC TCT C

K = G/T, R = G/A, Y = T/C, V = G/C/A, H = A/C/T for 5′-anchored SSR primer sequences.

**Table 3:** Different units of anchored primer and adapter primer.

	PAC	PCT	Total
Adapter 1	2	6	8^b^
Adapter 2	3	5	8^b^
Adapter 3	3	4	7^b^
Adapter 4	1	3	4^b^
Adapter 5	5	6	11^b^
Adapter 6	3	3	6^b^
Adapter 7	3	3	6^b^
Adapter 8	2	4	6^b^
Adapter 9	1	4	5^b^
Adapter 10	4	4	8^b^
Adapter 11	5	3	8^b^
Adapter 12	3	6	9^b^
Total	35^a^	51^a^	86^c^

^a^Number of sequences derived from the units of different *PstI* adapter and two 5′-anchor primers.

^b^Number of sequences derived from the units of twelve *PstI *adapters and different 5′-anchor primers.

^c^Number of sequences derived from the units of twelve *PstI *adapters and two 5′-anchor primers.

**Table 4:** Frequency and distribution of SSRs in the analysed nonredundant 1110 pineapple ESTs.

Repeats motif	Number of repeat units											Total repeats
	5	6	7	8	9	10	11	12	14	15	>16	
A/T	—	—	—	—	—	149	87	67	35	26	100	511
C/G	—	—	—	—	—	5	1	1	2			11
AC/GT	—	8	4	1	1	2	2		1		3	23
AG/CT	—	54	34	40	19	25	19	16	19	14	60	318
AT/AT	—	17	4	9	5	3		1				39
CG/CG	—		1									1
AAC/GTT	—	2	1	1	2		1		1			8
AAG/CTT	—	14	8	7		2	1					33
AAT/ATT	—	3	6	1	2	2						14
ACC/GGT	—	1	2	1		1						5
ACG/CTG	—	6	6	2	2	1	1	1				19
ACT/ATG	—	3	2	1				2				8
AGC/CGT	—	10	6	5	6	2	8					38
AGG/CCT	—	14	8	2								24
AGT/ATC	—		2									2
CCG/CGG	—	3	1		2	1						7
AAAC/GTTT		1										1
AAAG/CTTT	5	2	1									8
AAAT/ATTT	1			1								2
AATC/AGTT	1											1
ACGT/ATGC		1										1
ACTG/ACTG					1							1
AGAT/ATCT	1											1
AGCC/CGGT		1										1
AGCG/CGCT	2											2
AGCT/ATCG	1											1
AGGT/ATCC				4								4
AAAAG/CTTTT	3	1										4
AAAAT/ATTTT	2	1										3
AACAC/GTGTT	1											1
AAGAG/CTCTT	1											1
AATCG/AGCTT	1											1
ACCAT/ATGGT	1											1
AGCCG/CGGCT	1											1
AAAAAG/CTTTTT	3											3
AACCCT/ATTGGG	1											1
AACTAC/ATGTTG	1											1
AAGAGG/CCTTCT		1										1
AAGGAG/CCTCTT	2											2
AAGGCG/CCGCTT	2											2
AATCCC/AGGGTT	1											1
ACGGCG/CCGCTG		1										1
AGCAGG/CCTCGT	1											1
AGCGTC/AGTCGC			1									1

**Table 5:** Details of the SSR primers for genetic diversity analysis.

Prime Pairs	Sequence ID	SSR motif	Forward primer (5′-3′)	Reverse primer (5′-3′)	Expected product size (bp)
Bp-01	AC 1.3	(CA)8	TCACACACACACACACAAAAAC	ATGGATTGCGCTGAGCTG	119
Bp-08	AC 6.3	(TG)8	ATGATGCCAGTGGAGTGTTC	ACACACACACACTTTTCTCATTG	152
EP-02	DT339694	(GAA)7	CGTGCCGCATAAATCAT	TATCTCCTCGCTCCTCTTG	116
EP-05	DT339172	(CCAT)8*⋯* (AT)7	CAGCCAATAACAACCTCAAG	TCCATACACACAGTACGTCG	263
EP-06	DT339094	(CTTTTT)5	CGACTCGAGGATTACATTACG	GAGCACAAAGAACCACACAG	270
EP-09	DT338799	(TC)19	CCGAGGAAGAAGAAGAGGT	GGTCCACAGTTGTTTCAGTT	160
EP-10	DT338783	(GAT)7	GACCTTTATCCATCGCATC	CCATCAAACGTGAAATCTTG	266
EP-11	DT338752	(CAGGAG)5	AGCGAGATAGCAGAGATAGG	TAGAGCGATGTTCGGATG	180
EP-12	DT338506	(AG)6*⋯* (AG)6	TTAACACATGCACGGAGTAC	CTAAGAGACAACCCAGGAAG	236
EP-13	DT338494	(CCAT)8*⋯* (AT)8	GCCAATAACAACCTCAAGC	TCCATACACACAGTACGTCG	263
EP-15	DT338171	(GCAGTC)7	ACCTACAAGTGGTACGTCG	GGAGCAAGGAGTTATTCAG	242
EP-16	DT338171	(TC)6*⋯* (AG)18	TAGTGAGTCAGGAGGAGAATG	CAAATAAACGGAGCGGAT	212
EP-20	DT337383	(TCT)8	TAATCGGGTGGAGTAAGG	GCTCACATAGGCCAATATG	155
EP-23	DT337096	(TC)20	ATGGTGGTTCACTTATCAGC	AGACATTCAAAGCGGAGAG	126
EP-24	DT337054	(CT)10	GCTGCTCTTGCTGCCAT	AAGCCATAGGACCACCAC	166
EP-26	DT336292	(AT)8	GAAGCGCAGGTTCGTAAT	ACAGAAGTAGAGGAAAGCAGC	227
EP-27	DT336032	(TCT)6	ATACTCTGCTGCTGTGAACG	TTGCACTCCTCTTTGCTAAC	155
EP-29	CO731867	(AGC)9	GCGAGCCTGTTAGACTTTGT	ACGATCTCAGCTGGACCTT	213

**Table 6:** Comparisons of different search criteria and software for SSR development.

	References		
	Wöhrmann and Weising [6]	Ong et al. [19]	In this study
Software to assemble ESTs	Geneious 5.0 software	SeqMan software	StackPACK v2.2 program
Software to search SSRs	SciROKO	SynaRex tool	MISA
Search criteria	Mono- ≥ 15, di- ≥ 7, tri-, tetra-, penta-, hexa- ≥ 5	Di- ≥ 8, tri- ≥ 6, tetra- ≥ 5	Mono- ≥ 10, di-, tri- ≥ 6, tetra-, penta-, hexa- ≥ 5
No. of ESTs analysised	5659 Moyle et al. [20]	5931 Moyle et al. [20], Ong et al. [19]	5659 Moyle et al. [20]
No. of SSRs identified	581	416	588
No. of SSR motif types	42	5	44
Frequency of SSRs	1/4.1 KB	Not mentioned	1/3.73 KB
SSRs for primerdesign	537	133	588
No. of di-	240	203	381
No. of tri-	251	213	158
No. of (CG)n	2	0	1

Mono-, di-, tri-, tetra-, penta- and hexa- represent mononucleotide repeats, dinucleotide repeats, trinucleotide repeats, tetranucleotide repeats and hexanucleotide repeats, respectively.
